# Di-μ_3_-chlorido-tetra-μ_2_-chlorido-dichloridobis(dimethyl­formamide-κ*O*)hexa­kis­(1*H*-imidazole-κ*N* ^3^)tetra­cadmium

**DOI:** 10.1107/S1600536811037834

**Published:** 2011-09-30

**Authors:** Run-Qiang Zhu

**Affiliations:** a Ordered Matter Science Research Center, College of Chemistry and Chemical Engineering, Southeast University, Nanjing 211189, People’s Republic of China

## Abstract

The centrosymmetric mol­ecule of the title complex, [Cd_4_Cl_8_(C_3_H_4_N_2_)_6_(C_3_H_7_NO)_2_], contains four Cd^II^ atoms, six imidazole, two dimethyl­formamide and eight chloride ligands. The structure shows a novel chloride-bridged tetra­nuclear cadmium quasi-cubane cluster. The coordination geometry of all Cd^II^ atoms is distorted octa­hedral, with the two metal atoms in the asymmetric unit in different coordination environments. One of the Cd^2+^ ions is coordinated by five Cl^−^ ions and by one N atom from an imidazole ligand, while the second is coordinated by three chloride ligands, two N atoms from two imidazole ligands and one O atom from a dimethyl­formamide mol­ecule. Inter­molecular N—H⋯Cl hydrogen bonds link the mol­ecules into a two-dimensional polymeric structure parallel to the *ab* plane.

## Related literature

For general background to ferroelectric compounds with metal-organic frameworks, see: Ye *et al.* (2009 ▶); Zhang *et al.* (2009 ▶).
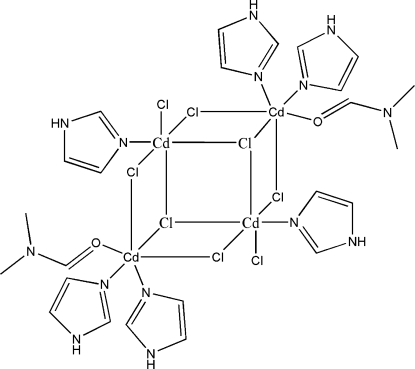


## Experimental

### 

#### Crystal data

[Cd_4_Cl_8_(C_3_H_4_N_2_)_6_(C_3_H_7_NO)_2_]
*M* *_r_* = 1287.92
Monoclinic, 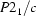

*a* = 8.2540 (17) Å
*b* = 12.290 (3) Å
*c* = 21.119 (4) Å
β = 99.23 (3)°
*V* = 2114.6 (8) Å^3^
*Z* = 2
Mo *K*α radiation
μ = 2.53 mm^−1^
*T* = 293 K
0.30 × 0.25 × 0.20 mm

#### Data collection

Rigaku SCXmini diffractometer
Absorption correction: multi-scan (*CrystalClear*; Rigaku, 2005 ▶) *T*_min_ = 0.472, *T*_max_ = 0.603
21502 measured reflections
4833 independent reflections
4241 reflections with *I* > 2σ(*I*)
*R*_int_ = 0.037

#### Refinement

*R*[*F* ^2^ > 2σ(*F* ^2^)] = 0.028
*wR*(*F* ^2^) = 0.069
*S* = 1.05
4833 reflections
237 parameters
H-atom parameters constrained
Δρ_max_ = 0.34 e Å^−3^
Δρ_min_ = −0.66 e Å^−3^

### 

Data collection: *CrystalClear* (Rigaku, 2005 ▶); cell refinement: *CrystalClear*; data reduction: *CrystalClear*; program(s) used to solve structure: *SHELXS97* (Sheldrick, 2008 ▶); program(s) used to refine structure: *SHELXL97* (Sheldrick, 2008 ▶); molecular graphics: *DIAMOND* (Brandenburg & Putz, 2005 ▶); software used to prepare material for publication: *SHELXL97*.

## Supplementary Material

Crystal structure: contains datablock(s) I, global. DOI: 10.1107/S1600536811037834/gk2402sup1.cif

Structure factors: contains datablock(s) I. DOI: 10.1107/S1600536811037834/gk2402Isup2.hkl

Additional supplementary materials:  crystallographic information; 3D view; checkCIF report

## Figures and Tables

**Table 1:** Hydrogen-bond geometry (Å, °)

*D*—H⋯*A*	*D*—H	H⋯*A*	*D*⋯*A*	*D*—H⋯*A*
N2—H2*A*⋯Cl2^i^	0.86	2.44	3.226 (3)	152
N4—H4*A*⋯Cl1^ii^	0.86	2.45	3.212 (3)	148
N6—H6*A*⋯Cl2^iii^	0.86	2.63	3.314 (3)	137

Symmetry codes: (i) 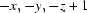; (ii) 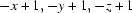; (iii) 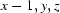.

**Fig. 1.**
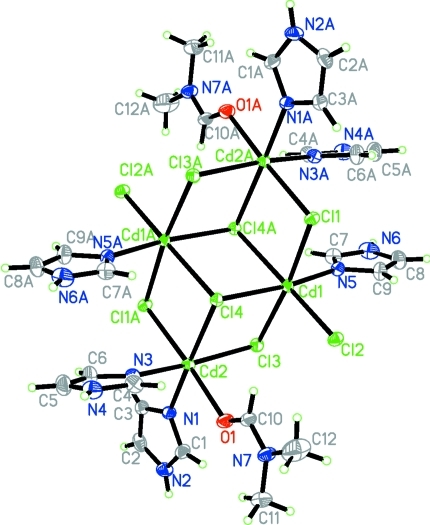
The molecular structure of the title complex with displacement ellipsoids shown at the 50% probability level. Symmetry codes for the atoms with the A label: -x, 1 - y, 1 - z.

**Fig. 2.**
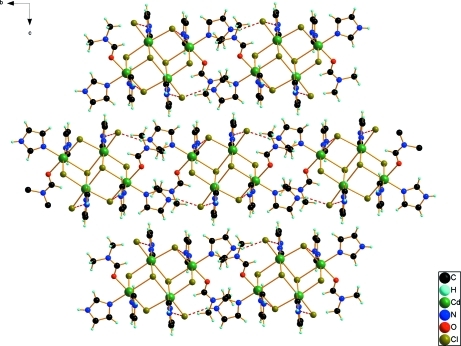
Packing diagram of the title compound projected along the a axis. Hydrogen bonds are shown as dashed lines.

## supplementary crystallographic information

### Comment

The title compound (I) was prepared from imidazole and cadmium(II) chloride in DMF. The solid state structure of (I) at 298 K shows a novel centrosymmetric tetranuclear cadmium quasi-cubane cluster with a Cd_4_(Cl)_2_(µ-Cl)_4_(µ_3_-Cl)_2_ core structure surrounded by six imidazole and two DMF molecules (Fig. 1). There are two different coordination environments about the Cd centers: Cd(1) is coordinated by one imidazole ligand, four bridging and one terminal Cl ions, and Cd(2) is coordinated by one O atom from DMF, two N atoms from two imidazole ligands and three bridging Cl ions. The shortest intra-molecular Cd(1)—Cd(1 A) separation is 4.103 (5) Å.

In the tetranuclear cluster, the cadmium atoms are connected by six Cl atoms among which the Cl1 and Cl3 atoms act as bridges between Cd(1) and Cd(2) centers, and the Cl(4) atom is a node to connect two Cd1 and one Cd2 together. The bond length Cd1–Cl2 to the terminal Cl ligand of 2.5475 (10) Å is shorter than the mean values of Cd–Cl(µ) [2.654 (2) Å] and Cd—Cl(µ3) [2.729 (2) Å] bond lengths.

In order to check a possibility of a structural phase transitions in compound (I), we measured its temperature-dependent dielectric constant. Large dielectric anomalies usually indicate structural changes such as paraelectric-to-ferroelectric phase transitions. Unfortunately, the dielectric constant of compound (I) goes smoothly in the temperature range 93–273 K, suggesting no distinct phase transitions occurring in this temperature range (Ye *et al.*, 2009; Zhang *et al.*, 2009).

### Experimental

The mixture of CdCl_2_ (2.27 g, 10 mmol) and imidazole (2.76 g, 40 mmol) in DMF was stirred for several days at room temperature. Colourless needle-like crystals suitable for X-ray diffraction analysis were obtained by slow evaporation of the solution at room temperature over 2 weeks.

### Refinement

Positional parameters of all H atoms were calculated geometrically and the H atoms were set to ride on the C atoms and N atoms to which they were bonded, with *U*_iso_(H)= 1.2 *U*_iso_(C, N) and 1.5*U*_iso_(C) for methyl H atoms. C—H atoms were included with bond distances ranging from 0.98 to 1.00 Å and N—H hydrogen atoms were included with the N–H distance set to 0.84 Å.

### Crystal data

**Table:**

[Cd_4_Cl_8_(C_3_H_4_N_2_)_6_(C_3_H_7_NO)_2_]	*F*(000) = 1248
*M**_r_* = 1287.92	*D*_x_ = 2.023 Mg m^−^^3^
Monoclinic, *P*2_1_/*c*	Mo *K*α radiation, λ = 0.71073 Å
Hall symbol: -P 2ybc	Cell parameters from 4835 reflections
*a* = 8.2540 (17) Å	θ = 2.5–27.5°
*b* = 12.290 (3) Å	µ = 2.53 mm^−^^1^
*c* = 21.119 (4) Å	*T* = 293 K
β = 99.23 (3)°	Prism, colourless
*V* = 2114.6 (8) Å^3^	0.30 × 0.25 × 0.20 mm
*Z* = 2	

### Data collection

**Table:**

Rigaku SCXmini diffractometer	4833 independent reflections
Radiation source: fine-focus sealed tube	4241 reflections with *I* > 2σ(*I*)
graphite	*R*_int_ = 0.037
CCD_Profile_fitting scans	θ_max_ = 27.5°, θ_min_ = 3.0°
Absorption correction: multi-scan (*CrystalClear*; Rigaku, 2005)	*h* = −10→10
*T*_min_ = 0.472, *T*_max_ = 0.603	*k* = −15→15
21502 measured reflections	*l* = −27→27

### Refinement

**Table:**

Refinement on *F*^2^	Secondary atom site location: difference Fourier map
Least-squares matrix: full	Hydrogen site location: inferred from neighbouring sites
*R*[*F*^2^ > 2σ(*F*^2^)] = 0.028	H-atom parameters constrained
*wR*(*F*^2^) = 0.069	*w* = 1/[σ^2^(*F*_o_^2^) + (0.0349*P*)^2^ + 1.0319*P*] where *P* = (*F*_o_^2^ + 2*F*_c_^2^)/3
*S* = 1.05	(Δ/σ)_max_ = 0.002
4833 reflections	Δρ_max_ = 0.34 e Å^−^^3^
237 parameters	Δρ_min_ = −0.66 e Å^−^^3^
0 restraints	Extinction correction: *SHELXL97* (Sheldrick, 2008)
Primary atom site location: structure-invariant direct methods	Extinction coefficient: 0

### Special details

**Table:**

Geometry. All e.s.d.'s (except the e.s.d. in the dihedral angle between two l.s. planes) are estimated using the full covariance matrix. The cell e.s.d.'s are taken into account individually in the estimation of e.s.d.'s in distances, angles and torsion angles; correlations between e.s.d.'s in cell parameters are only used when they are defined by crystal symmetry. An approximate (isotropic) treatment of cell e.s.d.'s is used for estimating e.s.d.'s involving l.s. planes.
Refinement. Refinement of *F*^2^ against ALL reflections. The weighted *R*-factor *wR* and goodness of fit *S* are based on *F*^2^, conventional *R*-factors *R* are based on *F*, with *F* set to zero for negative *F*^2^. The threshold expression of *F*^2^ > σ(*F*^2^) is used only for calculating *R*-factors(gt) *etc*. and is not relevant to the choice of reflections for refinement. *R*-factors based on *F*^2^ are statistically about twice as large as those based on *F*, and *R*- factors based on ALL data will be even larger.

### Fractional atomic coordinates and isotropic or equivalent isotropic displacement parameters (Å^2^)

**Table:**

	*x*	*y*	*z*	*U*_iso_*/*U*_eq_	
C1	0.1022 (4)	0.0007 (3)	0.59318 (17)	0.0415 (8)	
H1	0.1309	−0.0205	0.5542	0.050*	
C2	0.0277 (4)	−0.0083 (3)	0.68722 (18)	0.0457 (9)	
H2	−0.0036	−0.0348	0.7247	0.055*	
C3	0.0450 (4)	0.0970 (3)	0.67154 (16)	0.0380 (8)	
H3	0.0275	0.1563	0.6969	0.046*	
C4	0.5413 (4)	0.2881 (3)	0.57582 (18)	0.0416 (8)	
H4	0.5389	0.2891	0.5316	0.050*	
C5	0.6345 (5)	0.2923 (4)	0.6781 (2)	0.0559 (10)	
H5	0.7043	0.2964	0.7172	0.067*	
C6	0.4692 (4)	0.2792 (3)	0.66852 (18)	0.0484 (9)	
H6	0.4053	0.2730	0.7008	0.058*	
C7	−0.5008 (4)	0.4395 (3)	0.38982 (19)	0.0429 (8)	
H7	−0.5052	0.4478	0.4333	0.051*	
C8	−0.5764 (5)	0.4230 (3)	0.2874 (2)	0.0582 (11)	
H8	−0.6402	0.4178	0.2470	0.070*	
C9	−0.4130 (5)	0.4196 (3)	0.30070 (17)	0.0457 (9)	
H9	−0.3428	0.4114	0.2707	0.055*	
C10	0.3020 (4)	0.1635 (3)	0.43669 (16)	0.0384 (8)	
H10	0.2958	0.2363	0.4245	0.046*	
C11	0.3797 (6)	−0.0201 (3)	0.4151 (2)	0.0638 (12)	
H11A	0.3284	−0.0347	0.4519	0.096*	
H11B	0.4930	−0.0415	0.4239	0.096*	
H11C	0.3249	−0.0605	0.3790	0.096*	
C12	0.4289 (8)	0.1321 (5)	0.3439 (3)	0.096 (2)	
H12A	0.3740	0.0932	0.3072	0.144*	
H12B	0.5449	0.1192	0.3485	0.144*	
H12C	0.4078	0.2086	0.3382	0.144*	
Cd1	−0.10174 (3)	0.428101 (17)	0.415237 (10)	0.02728 (7)	
Cd2	0.14449 (3)	0.254846 (17)	0.557490 (11)	0.02742 (7)	
N1	0.0926 (3)	0.1030 (2)	0.61219 (12)	0.0322 (6)	
N2	0.0649 (4)	−0.0674 (2)	0.63767 (16)	0.0480 (8)	
H2A	0.0647	−0.1373	0.6353	0.058*	
N3	0.4108 (3)	0.2763 (2)	0.60408 (13)	0.0319 (6)	
N4	0.6769 (4)	0.2984 (3)	0.61908 (17)	0.0502 (8)	
H4A	0.7747	0.3073	0.6108	0.060*	
N5	−0.3653 (3)	0.4301 (2)	0.36550 (13)	0.0318 (6)	
N6	−0.6315 (4)	0.4356 (3)	0.34380 (19)	0.0549 (9)	
H6A	−0.7325	0.4402	0.3490	0.066*	
N7	0.3690 (4)	0.0952 (2)	0.40063 (15)	0.0433 (7)	
O1	0.2472 (3)	0.13812 (19)	0.48519 (11)	0.0423 (6)	
Cl1	−0.04823 (9)	0.61386 (6)	0.35684 (4)	0.02980 (16)	
Cl2	0.00339 (10)	0.31740 (6)	0.32819 (4)	0.03436 (17)	
Cl3	−0.14308 (9)	0.25405 (6)	0.48437 (4)	0.03149 (17)	
Cl4	0.19278 (8)	0.44090 (6)	0.48833 (3)	0.02730 (15)	

### Atomic displacement parameters (Å^2^)

**Table:**

	*U*^11^	*U*^22^	*U*^33^	*U*^12^	*U*^13^	*U*^23^
C1	0.053 (2)	0.0290 (17)	0.042 (2)	−0.0073 (16)	0.0058 (16)	−0.0043 (15)
C2	0.045 (2)	0.052 (2)	0.038 (2)	−0.0087 (17)	0.0011 (16)	0.0146 (17)
C3	0.0392 (18)	0.0418 (19)	0.0326 (18)	−0.0001 (15)	0.0045 (14)	0.0006 (15)
C4	0.0326 (18)	0.046 (2)	0.048 (2)	0.0014 (16)	0.0132 (15)	0.0057 (17)
C5	0.043 (2)	0.066 (3)	0.054 (3)	−0.002 (2)	−0.0067 (18)	−0.003 (2)
C6	0.041 (2)	0.065 (3)	0.040 (2)	−0.0048 (18)	0.0077 (16)	−0.0007 (18)
C7	0.0342 (18)	0.044 (2)	0.052 (2)	−0.0048 (15)	0.0136 (16)	0.0018 (16)
C8	0.040 (2)	0.067 (3)	0.059 (3)	0.001 (2)	−0.0162 (19)	−0.002 (2)
C9	0.042 (2)	0.062 (2)	0.0334 (19)	−0.0014 (18)	0.0045 (15)	−0.0018 (17)
C10	0.047 (2)	0.0311 (17)	0.0377 (19)	0.0053 (15)	0.0091 (15)	−0.0054 (14)
C11	0.078 (3)	0.042 (2)	0.072 (3)	0.019 (2)	0.014 (2)	−0.007 (2)
C12	0.143 (5)	0.082 (4)	0.080 (4)	0.007 (4)	0.071 (4)	0.001 (3)
Cd1	0.02655 (12)	0.02762 (12)	0.02745 (13)	−0.00098 (9)	0.00362 (9)	−0.00129 (9)
Cd2	0.02759 (12)	0.02543 (12)	0.03022 (13)	0.00193 (9)	0.00767 (9)	0.00044 (9)
N1	0.0365 (14)	0.0279 (13)	0.0323 (15)	−0.0019 (11)	0.0058 (11)	0.0029 (11)
N2	0.0547 (19)	0.0268 (15)	0.060 (2)	−0.0064 (14)	0.0004 (16)	0.0056 (14)
N3	0.0283 (13)	0.0317 (14)	0.0369 (15)	0.0022 (11)	0.0090 (11)	0.0024 (11)
N4	0.0294 (16)	0.0476 (19)	0.074 (2)	−0.0023 (14)	0.0098 (15)	0.0005 (17)
N5	0.0266 (13)	0.0353 (14)	0.0334 (15)	−0.0027 (11)	0.0047 (11)	−0.0015 (11)
N6	0.0235 (15)	0.053 (2)	0.088 (3)	−0.0017 (14)	0.0089 (16)	0.0052 (18)
N7	0.0524 (18)	0.0368 (16)	0.0440 (17)	0.0076 (14)	0.0178 (14)	−0.0062 (13)
O1	0.0542 (15)	0.0369 (13)	0.0385 (14)	0.0081 (11)	0.0156 (11)	−0.0038 (10)
Cl1	0.0327 (4)	0.0265 (4)	0.0323 (4)	−0.0034 (3)	0.0117 (3)	−0.0020 (3)
Cl2	0.0418 (4)	0.0305 (4)	0.0335 (4)	−0.0011 (3)	0.0144 (3)	−0.0021 (3)
Cl3	0.0327 (4)	0.0306 (4)	0.0313 (4)	−0.0037 (3)	0.0057 (3)	0.0020 (3)
Cl4	0.0249 (3)	0.0298 (4)	0.0277 (4)	0.0005 (3)	0.0058 (3)	−0.0008 (3)

### Geometric parameters (Å, °)

**Table:**

C1—N1	1.326 (4)	C10—H10	0.9300
C1—N2	1.330 (5)	C11—N7	1.450 (5)
C1—H1	0.9300	C11—H11A	0.9600
C2—C3	1.349 (5)	C11—H11B	0.9600
C2—N2	1.350 (5)	C11—H11C	0.9600
C2—H2	0.9300	C12—N7	1.440 (5)
C3—N1	1.374 (4)	C12—H12A	0.9600
C3—H3	0.9300	C12—H12B	0.9600
C4—N3	1.320 (4)	C12—H12C	0.9600
C4—N4	1.332 (5)	Cd1—N5	2.259 (3)
C4—H4	0.9300	Cd1—Cl2	2.5486 (9)
C5—N4	1.349 (5)	Cd1—Cl3	2.6426 (9)
C5—C6	1.357 (5)	Cd1—Cl1	2.6651 (9)
C5—H5	0.9300	Cd1—Cl4	2.6671 (11)
C6—N3	1.369 (4)	Cd1—Cl4^i^	2.7920 (9)
C6—H6	0.9300	Cd2—N1	2.272 (3)
C7—N5	1.308 (4)	Cd2—N3	2.275 (3)
C7—N6	1.332 (5)	Cd2—O1	2.350 (2)
C7—H7	0.9300	Cd2—Cl3	2.6158 (12)
C8—C9	1.333 (5)	Cd2—Cl1^i^	2.6400 (9)
C8—N6	1.350 (6)	Cd2—Cl4	2.7762 (9)
C8—H8	0.9300	N2—H2A	0.8600
C9—N5	1.368 (4)	N4—H4A	0.8600
C9—H9	0.9300	N6—H6A	0.8600
C10—O1	1.225 (4)	Cl1—Cd2^i^	2.6400 (9)
C10—N7	1.313 (4)	Cl4—Cd1^i^	2.7920 (9)
			
N1—C1—N2	110.5 (3)	Cl3—Cd1—Cl4	85.09 (3)
N1—C1—H1	124.7	Cl1—Cd1—Cl4	90.80 (3)
N2—C1—H1	124.7	N5—Cd1—Cl4^i^	88.92 (7)
C3—C2—N2	106.3 (3)	Cl2—Cd1—Cl4^i^	175.27 (2)
C3—C2—H2	126.9	Cl3—Cd1—Cl4^i^	89.43 (3)
N2—C2—H2	126.9	Cl1—Cd1—Cl4^i^	85.84 (3)
C2—C3—N1	109.4 (3)	Cl4—Cd1—Cl4^i^	82.58 (3)
C2—C3—H3	125.3	N1—Cd2—N3	97.08 (9)
N1—C3—H3	125.3	N1—Cd2—O1	86.90 (9)
N3—C4—N4	110.9 (3)	N3—Cd2—O1	85.85 (9)
N3—C4—H4	124.6	N1—Cd2—Cl3	94.01 (7)
N4—C4—H4	124.6	N3—Cd2—Cl3	167.97 (7)
N4—C5—C6	105.8 (3)	O1—Cd2—Cl3	90.10 (7)
N4—C5—H5	127.1	N1—Cd2—Cl1^i^	92.97 (7)
C6—C5—H5	127.1	N3—Cd2—Cl1^i^	90.51 (7)
C5—C6—N3	109.6 (3)	O1—Cd2—Cl1^i^	176.31 (6)
C5—C6—H6	125.2	Cl3—Cd2—Cl1^i^	93.58 (3)
N3—C6—H6	125.2	N1—Cd2—Cl4	177.38 (7)
N5—C7—N6	110.7 (3)	N3—Cd2—Cl4	85.51 (7)
N5—C7—H7	124.6	O1—Cd2—Cl4	93.66 (6)
N6—C7—H7	124.6	Cl3—Cd2—Cl4	83.44 (3)
C9—C8—N6	106.9 (4)	Cl1^i^—Cd2—Cl4	86.65 (3)
C9—C8—H8	126.5	C1—N1—C3	105.4 (3)
N6—C8—H8	126.5	C1—N1—Cd2	126.9 (2)
C8—C9—N5	109.0 (4)	C3—N1—Cd2	127.7 (2)
C8—C9—H9	125.5	C1—N2—C2	108.4 (3)
N5—C9—H9	125.5	C1—N2—H2A	125.8
O1—C10—N7	124.7 (3)	C2—N2—H2A	125.8
O1—C10—H10	117.7	C4—N3—C6	105.3 (3)
N7—C10—H10	117.7	C4—N3—Cd2	128.2 (2)
N7—C11—H11A	109.5	C6—N3—Cd2	126.5 (2)
N7—C11—H11B	109.5	C4—N4—C5	108.3 (3)
H11A—C11—H11B	109.5	C4—N4—H4A	125.8
N7—C11—H11C	109.5	C5—N4—H4A	125.8
H11A—C11—H11C	109.5	C7—N5—C9	105.9 (3)
H11B—C11—H11C	109.5	C7—N5—Cd1	129.7 (2)
N7—C12—H12A	109.5	C9—N5—Cd1	124.4 (2)
N7—C12—H12B	109.5	C7—N6—C8	107.4 (3)
H12A—C12—H12B	109.5	C7—N6—H6A	126.3
N7—C12—H12C	109.5	C8—N6—H6A	126.3
H12A—C12—H12C	109.5	C10—N7—C12	120.9 (4)
H12B—C12—H12C	109.5	C10—N7—C11	121.3 (3)
N5—Cd1—Cl2	94.83 (7)	C12—N7—C11	117.7 (3)
N5—Cd1—Cl3	93.76 (7)	C10—O1—Cd2	127.5 (2)
Cl2—Cd1—Cl3	93.18 (3)	Cd2^i^—Cl1—Cd1	96.66 (3)
N5—Cd1—Cl1	89.68 (7)	Cd2—Cl3—Cd1	97.92 (3)
Cl2—Cd1—Cl1	91.30 (3)	Cd1—Cl4—Cd2	93.53 (3)
Cl3—Cd1—Cl1	174.11 (2)	Cd1—Cl4—Cd1^i^	97.42 (3)
N5—Cd1—Cl4	171.43 (7)	Cd2—Cl4—Cd1^i^	90.75 (3)
Cl2—Cd1—Cl4	93.71 (3)		

Symmetry codes: (i) −*x*, −*y*+1, −*z*+1.

### Hydrogen-bond geometry (Å, °)

**Table:**

*D*—H···*A*	*D*—H	H···*A*	*D*···*A*	*D*—H···*A*
N2—H2A···Cl2^ii^	0.86	2.44	3.226 (3)	152.
N4—H4A···Cl1^iii^	0.86	2.45	3.212 (3)	148.
N6—H6A···Cl2^iv^	0.86	2.63	3.314 (3)	137.

Symmetry codes: (ii) −*x*, −*y*, −*z*+1; (iii) −*x*+1, −*y*+1, −*z*+1; (iv) *x*−1, *y*, *z*.
